# Robustness of three external beam treatment techniques against inter‐fractional positional variations of the metal port in breast tissue expanders

**DOI:** 10.1002/acm2.13474

**Published:** 2021-11-22

**Authors:** Keren Mayorov, Patricia Lacasse, Elsayed Ali

**Affiliations:** ^1^ Department of Physics Carleton University Ottawa Ontario Canada; ^2^ The Ottawa Hospital Cancer Centre Ottawa Ontario Canada

**Keywords:** breast reconstruction, breast tissue expanders, inter‐fraction motion, postmastectomy radiation, robust delivery

## Abstract

**Introduction:**

Temporary breast tissue expanders contain a metal port that varies in position throughout the course of radiation treatments. The purpose of this study was to quantify the robustness of the three most common external beam treatment techniques (tangential three‐dimensional conformal radiation therapy [3DCRT], volumetric modulated arc therapy [VMAT], and helical tomotherapy) against our measured inter‐fractional positional variations of the port.

**Methods:**

For eight breast cases, a clinical plan was created for each of the three techniques. The dosimetric effect of our previously measured inter‐fractional port errors was evaluated for two classes of error: internal port errors (IPEs) and patient registration errors (PREs). For both classes of error, daily variable and systematic errors were modeled, and their cumulative effects were compared against the originally planned doses.

**Results:**

For systematic IPE, the 1%–99% range in point dose differences inside a 5‐mm target abutting the implant was the highest for tangential 3DCRT, and it was within 6% and 9% when calculated with Monte Carlo and collapsed cone calculation engines, respectively. Daily variable PRE resulted in mean changes of −3.0% and −3.5% to V_100%Rx_ of the target for VMAT and tomotherapy, respectively. For nearby organs, daily variable PRE resulted in changes to V_20Gy_ of the ipsilateral lung of less than 2% in all three techniques, while V_5Gy_ of the heart increased by as much as 6% in VMAT and 10% in tomotherapy.

**Conclusions:**

When IPEs were modeled, dose variability was the largest in tangential 3DCRT, leading to areas of underdosage in the shadow of the port. When PREs were modeled, the target coverage and nearby organs were affected the most in VMAT and helical tomotherapy. In reality, port positional errors result from a combination of IPE and PRE, suggesting that VMAT and tomotherapy are more robust when patient registration errors are minimized, despite the presence of IPE.

## 1 INTRODUCTION

For patients undergoing a postmastectomy breast reconstruction, a tissue expander is inserted at the time of the mastectomy to stretch the overlaying skin. The most common tissue expander contains a metal port made of a rare earth alloy magnet encapsulated in a titanium shell. The port is localized externally and used as an injection site for saline solution for gradual expansions. Some patients receive a radiation treatment while the temporary tissue expander is still in place, with the metal port in the field of radiation. The dosimetric effect of the static presence of the metal port during radiation therapy has been studied by other investigators, reporting an array of results.^1^, ^2^, ^3^, ^4^, ^5^, ^6^, ^7^, ^8^, ^9^, ^10^, ^11^, ^12^, ^13^ Our earlier study was the first to measure the inter‐fractional positional variations of the metal port.^14^ The earlier study analyzed data from treatments performed on helical tomotherapy because of the availability of daily megavoltage CT (MVCT) for image guidance. The reduced artefacts around the metal port in the MVCT images allowed for better localization of the port and for the quantification of its daily positional errors. However, helical treatments are carried out on a specialized linear accelerator that is not available in all centers. The two most common treatment techniques for breast radiation therapy are three‐dimensional conformal radiation therapy (3DCRT) and volumetric modulated arc therapy (VMAT), but the daily registration data they offer are limited in the context of the metal port.

In the current study, the previously measured errors in the position of the port were extrapolated to quantify and compare the robustness of the three most‐common techniques (tangential 3DCRT, VMAT and helical tomotherapy) for breast radiation treatment against inter‐fractional positional variations of the metal port. Relative changes in target coverage and doses to relevant organs at risk (OARs) were analyzed and compared for each of the three techniques when the measured port positional errors were simulated. The results of this study can inform appropriate planning strategies for more robust treatment delivery. This study differs from previous work in the literature by other investigators^1^, ^2^, ^3^, ^4^, ^5^, ^6^, ^7^, ^8^, ^9^, ^10^, ^11^, ^12^, ^13^ in that it addresses technique robustness against measured port positional errors rather than the dosimetric impact of the presence of a static metal port.

## 2 METHODS

### 2.1 Patient population

This study included eight anonymized postmastectomy breast cases as in our previous study, who received a radiation treatment on tomotherapy with the tissue expander in place. Three of the eight cases received a left‐sided breast irradiation. Each patient received 25 fractions to a total dose of 50 Gy, resulting in a total of 200 fractions, 193 of which were included in this study (seven fractions were not available due to un‐archiving issues).

### 2.2 Treatment planning

A clinical plan for each of the three techniques (3DCRT, VMAT, and helical tomotherapy) was created for each of the eight cases, for a total of 24 plans. The following is a brief summary of the treatment planning per technique.

For 3DCRT and VMAT plans, the commercial treatment planning system (TPS) Monaco 5.11 (Elekta AB, Sweden) was used to generate the plans. The beam model used was a 6 MV photon beam from an Elekta Synergy linear accelerator. The tangential 3DCRT plans consisted of two main half‐blocked parallel opposed fields that were open anteriorly for plan robustness against breathing motion during the treatment and against swelling of the breasts over the 5 weeks of treatment. Multiple smaller subfields were added to achieve a homogenous dose distribution. No wedges or bolus were used. The 3DCRT plans were calculated using both the collapsed cone (CC) and Monte Carlo (MC) dose calculation engines in Monaco. The MC calculations were performed to 1% uncertainty per control point, corresponding to an approximate uncertainty of 0.7% per plan.

The VMAT plans consisted of a single 230° arc with the isocenter located approximately in the center of the planning target volume (PTV). The PTV was defined as the 5‐mm expansion of the clinical extent of the chest wall, including the expander implant and its contents. The PTV was limited in the skin anteriorly and the ribs posteriorly. The target objectives were D_90%_ > 50.0 Gy and D_2%_ < 55.0 Gy. For the OARs, the dose constraints for the ipsilateral lung were D_mean_ < 16 Gy, V_5Gy_ < 50%, V_20Gy_ < 25%, and V_40Gy_ < 10%. For the heart, the constraints were D_mean_ < 8 Gy, V_5Gy_ < 50%, V_25Gy_ < 5%, and V_30Gy_ < 2.5%. For the VMAT plans, the MC‐based dose calculation algorithm was used. Similar to 3DCRT, the MC dose calculations were performed to 1% uncertainty per control point.

For helical tomotherapy, the original clinical plans were used since this cohort of patients was initially treated using this technique. The helical tomotherapy plans were calculated using the tomotherapy planning station, version 5.1.1.6. (Accuray, CA, USA) which implements convolution/superposition (CS) dose calculation engine.^15^

### 2.3 Modeling the measured inter‐fractional variations

The daily acquired MVCT images on tomotherapy have reduced artefacts around the metal port which allowed a more accurate localization of the port. The planned adaptive module of tomotherapy was used to load the co‐registered daily MVCT and the treatment CT, representing the true position of the patient relative to the planned position in each treatment fraction. In fractions where the metal port on the fused view of the MVCT and treatment planning CT did not align, the distance between the center of metal port on the two fused images was measured in the three cardinal directions. This distance is referred to herein as *port positional error*. From the previous study,^14^ the measured errors were generally small, with 87% of positional deviations smaller than 5 mm. However, the errors in the lateral, vertical, and longitudinal directions ranged from −17 to 11 mm, −10.8 to 7.0 mm and −8.0 to 7.0 mm, respectively.

Immobilization and positioning of patients for breast treatments at our center is identical in tangential 3DCRT, VMAT, and tomotherapy. However, tomotherapy is the only technique of the three that acquires daily MVCT images with reduced artefacts around the metal port. Thus, for the purpose of the current study, the inter‐fractional positional errors of the metal port that were measured for the cohort of patients treated on tomotherapy were assumed to apply to the tangential 3DCRT and VMAT treatment plans created for the same patients.

Our approach to assessing the dosimetric effect of the measured positional errors is by artificially modeling a shift in the position of the metal port in the TPS and comparing the resulted dose distribution against the originally planned dose. From the viewpoint of the metal port, the observed positional errors can be the caused by internal movement of the port, variations in daily patient setup, or a combination of the two. For modeling purposes, these causes were divided into two classes of error. The first class, referred to as internal port error (IPE), is the port displacement relative to the internal anatomy of the patient, caused by anatomical changes and/or the migration of the whole tissue expander. The second class, referred to as patient registration error (PRE), is the displacement of the whole patient relative to the treatment beam, caused by minor necessary compromises in the position of the patient during patient setup. These compromises are clinical judgments that are made every day when aligning the patient on the treatment couch before daily treatment.

**FIGURE 1:**
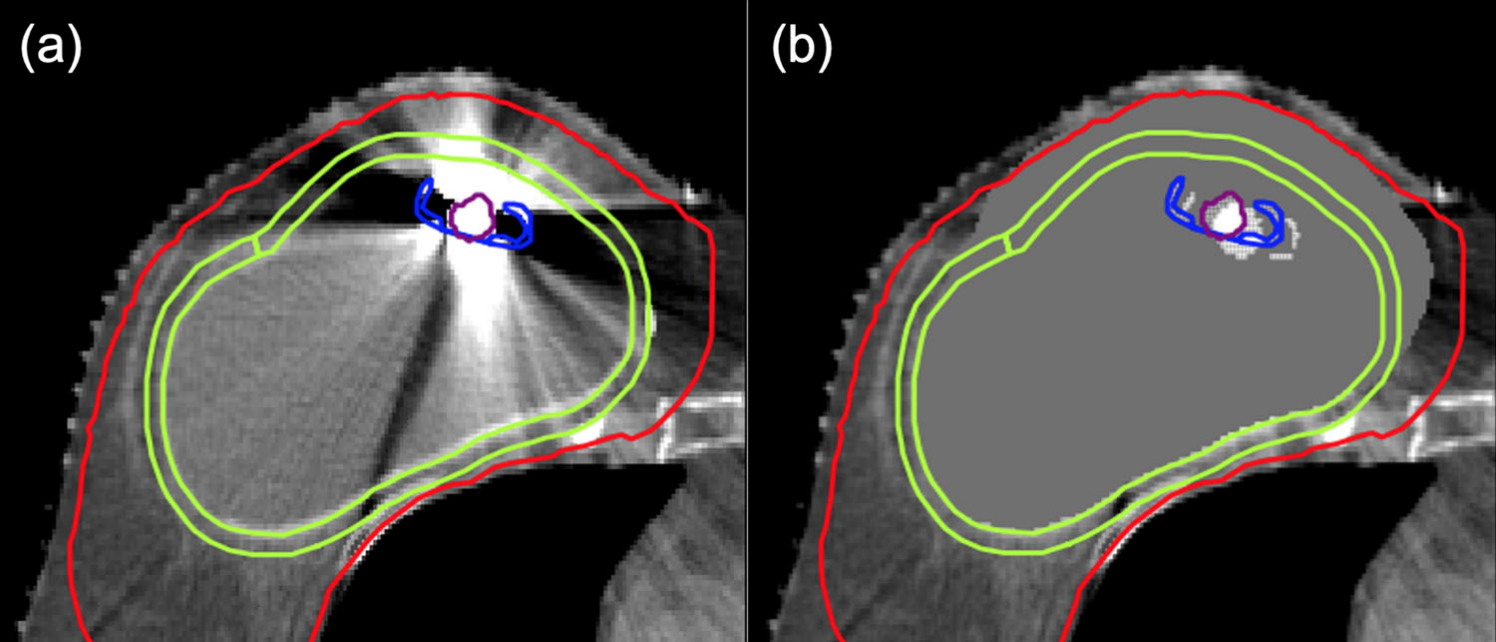
Computed Tomography (CT) slice of a representative patient before panel (a) and after panel (b) density overrides of the metal port and the tissue surrounding it. The maroon and blue contours are the magnetic core and titanium shell of the metal port, respectively. The area enclosed by the green contour is the region of interest (ROI), defined as a 5‐mm expansion around the saline implant. The red contour is the clinically defined planning target volume (PTV). The silhouette of the metal port in panel (b) demonstrates the artificially shifted metal port used for modeling internal port errors

To model IPE, the structure of the metal port was isolated in the treatment planning CT and artificially shifted by the magnitude of the measured daily displacement using an in‐house software developed for the purpose of this study. The original structure of the metal port contoured by the treatment planners during image segmentation was used. In slices with heavy metal artefacts in the treatment planning CT, the breast tissue around the metal port was overridden with a density value corresponding to the average breast density in artefact‐free slices. For the metal port, the nominal densities of the metal components of the port and shell were assigned. Voxels that were inside the titanium shell and the magnet were assigned Hounsfield unit values of 3926 and 10248, corresponding to physical densities of 4.0 g/cm^3^ and 8.0 g/cm^3^, respectively. An example of a corrected CT slice is shown in Figure 1. This was repeated for all fractions per patient, resulting in a separate CT set representing a given fraction, where the metal port is shifted from its original position by the measured error in that fraction. The data quality assurance features of Monaco and tomotherapy were used to calculate the tangential 3DCRT, VMAT, and tomotherapy plans on the modified CT sets of all fractions for all patients.

To model PRE, the metal port and the surrounding breast tissue in the original treatment planning CT were assigned density values as described above, with no displacements applied to the metal port. Within the data quality assurance feature of Monaco and tomotherapy, the corrected CT set of each patient was shifted relative to the planned photon fluence by the measured port displacement error of a given fraction, and the original plan of each respective technique was recalculated.

**FIGURE 2:**
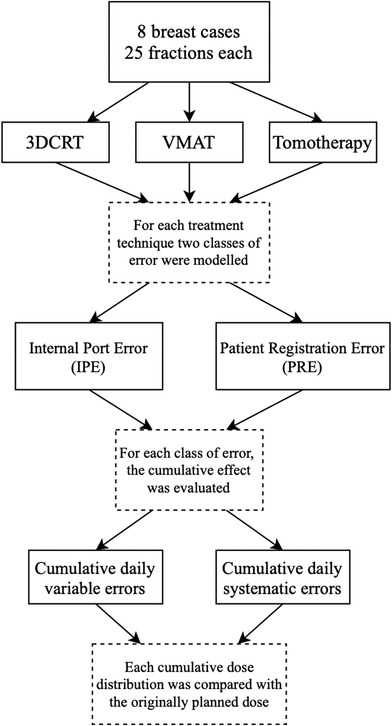
Flow chart summarizing the modeled scenarios of port positional errors included in the study. Internal port error (IPE) is displacement of the metal port relative to the internal anatomy of the patient. Patient registration error (PRE) is an error in the position of the port caused by a displacement of the whole patient relative to the radiation beam

For all eight breast cases, the dosimetric effects of IPE and PRE in each of the three treatment techniques (tangential 3DCRT, VMAT, and helical tomotherapy) were evaluated for two scenarios. The first scenario is the cumulative effect of the daily measured error, called in the results “daily variable” error, for which the calculated dose distributions for all fractions per patient were summed to a cumulative dose distribution. This scenario represents the true course of treatment. The second scenario is the cumulative effect of a systematic realistic large error in port position, called in the results “systematic” error. The magnitude of the error was derived from the largest positional error measured during metal port registration, and the same magnitude of error was simulated in all patients. The systematic error represents a change in port position after the treatment planning CT was acquired that persisted throughout the course of the treatment. For this scenario, the dose distribution of the plan with the simulated large error in port position was multiplied by the number of fractions to yield the equivalent of a cumulative dose distribution. For all three treatment techniques, the cumulative dose distributions were compared with the originally planned dose, that is, the clinical plan calculated on a density‐corrected CT set where neither the position of the metal port nor the position of the whole patient was changed. The flowchart in Figure 2 summarizes all modeled scenarios in the study.

In addition, the effect of ignoring the metal port during planning was compared between the three techniques for all patients. To model this, each plan was calculated on a corrected CT scan where the metal port was overridden with tissue‐equivalent density. The resulting dose distributions were compared with the originally planned dose, where the density of the metal port was included.

### 2.4 Robustness analysis

To quantify and compare the robustness of the three treatment techniques, point dose differences and dose volume histogram (DVH) parameters for a clinically meaningful region of interest (ROI) and for relevant OARs were calculated for all patients for the three treatment techniques. The ROI was defined as a 5‐mm expansion around the implant, provided that it is within the PTV (rationale below). The percent volume of the ROI receiving 100% of the dose (V_100%Rx_), the percent volume of the ipsilateral lung receiving 20 Gy (V_20Gy_), and the percent volume of the heart receiving 5 Gy (V_5Gy_) were evaluated. The analysis of the OARs was limited to the slices where the ROI was present.

The ROI used for robustness analysis was introduced because the clinically defined PTV contains the large non‐biological temporary implant, which can mask local changes to the dose distribution in clinically relevant areas. The expansion of the ROI was truncated to the limit of the clinical PTV. The ROI was defined in the slices where the metal port was present, including the farthest slices that the metal port migrated to during simulation. The newly defined ROI contains tissue directly abutting the implant, which is the more probable location of local recurrence.^16^

## 3 RESULTS

The perturbations to the overall dose distribution and the point dose differences are discussed first for their illustrative value, followed by the more clinically relevant changes in dose‐volume metrics.

### 3.1 Point dose differences

**FIGURE 3:**
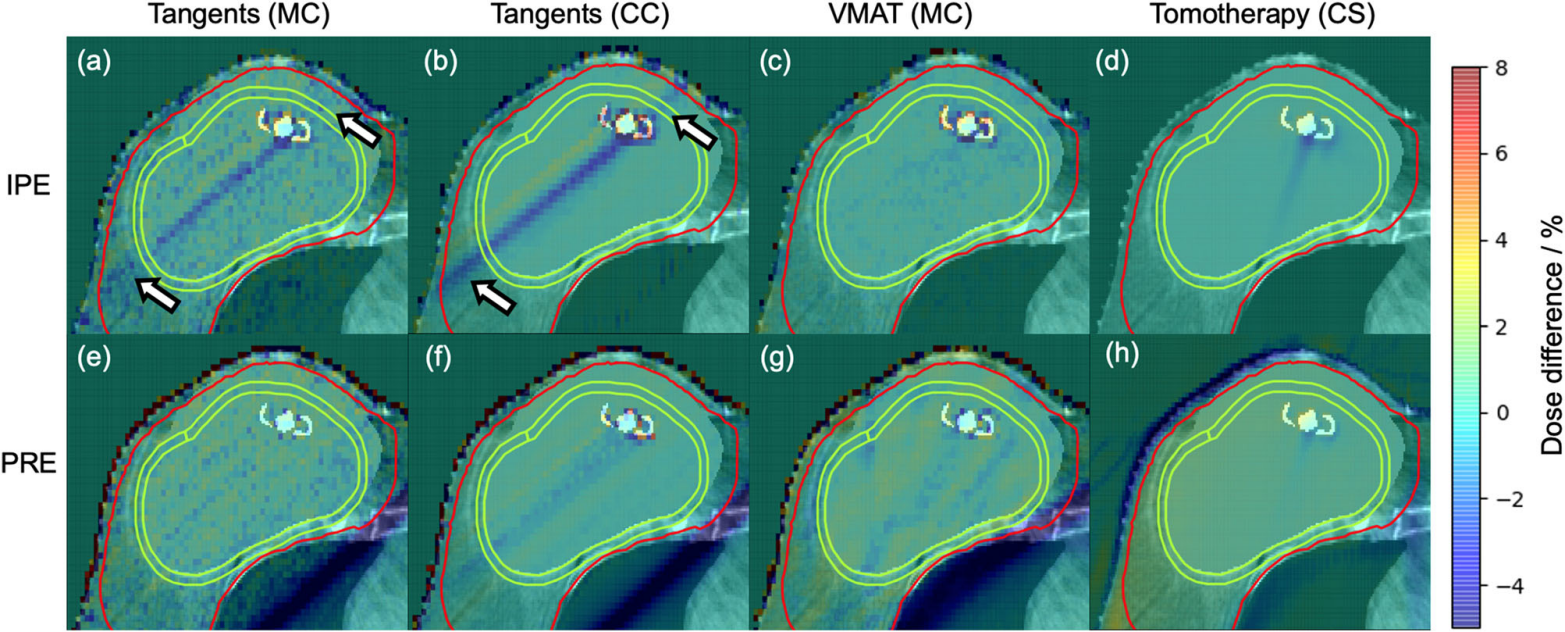
Percent dose difference maps for a representative slice and patient. Daily measured internal port error (IPE) is modeled in panels (a‐d), and daily measured patient registration error (PRE) is modeled in panels (e‐h). The dose calculation engine used with each treatment technique is shown in brackets. CC, collapsed cone; CS, convolution/superposition; MC, Monte Carlo. All percent dose differences are normalized to the prescription dose. The arrows in panel (a) and panel (b) point to areas of dose reduction outside the non‐biological tissue expander. The contours of the region of interest (ROI) and planning target volume (PTV) are defined as in Figure 1

Figure 3 shows the percent dose difference map of a representative patient relative to the original dose distribution when daily variable IPE and PRE were modeled in the three treatment techniques, all normalized to the prescription dose.

**FIGURE 4:**
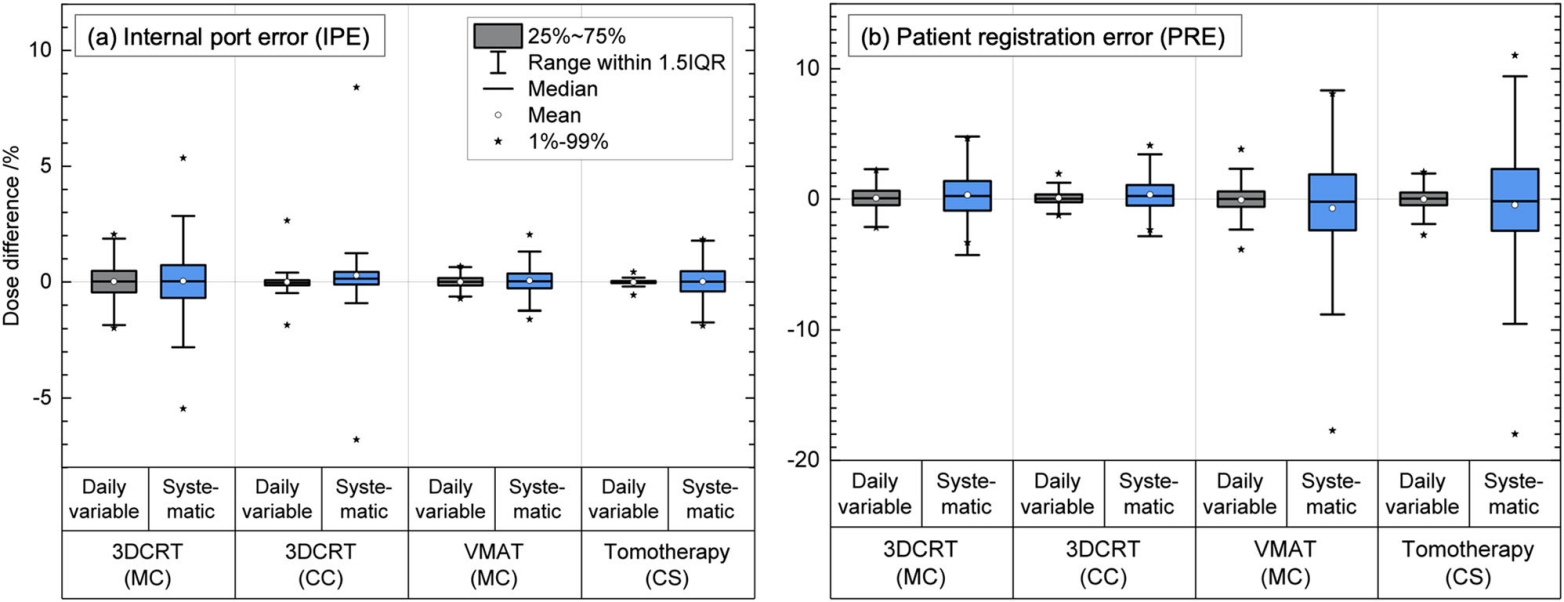
Percent point dose differences inside the region of interest (ROI) for all patients when port positional errors were modeled in the three treatment techniques (tangential three‐dimensional conformal radiation therapy [3DCRT], volumetric modulated arc therapy (VMAT), and helical tomotherapy). The modeled daily variable errors are in gray, and systematic errors are in blue. Panel (a) is for internal port error (IPE) and panel (b) is for patient registration error (PRE). All point dose differences were normalized to the prescription dose. The dose calculation engine used with each treatment technique is shown in brackets. CC, collapsed cone; CS, convolution/superposition; MC, Monte Carlo

The point dose differences inside the ROI for all patients when IPE (displacement of the metal port relative to the internal anatomy) was modeled is shown in the boxplot of Figure 4a. The cumulative effect of the daily variable and systematic IPE resulted in an absolute mean point dose differences close to zero, with the interquartile range being within 1% in all three techniques. The 1%–99% ranges for daily variable IPE were within 2% for 3DCRT (MC), 3% for 3DCRT (CC), 1% for VMAT (MC), and < 1% for tomotherapy (CS) treatment plans. For systematic IPE, the 1%–99% range in point dose differences was within 6% for 3DCRT (MC), 9% for 3DCRT (CC), 2% for VMAT (MC), and 2% for tomotherapy (CS).

The boxplot in Figure 4b shows the point dose differences inside the ROI for all patients when PREs (displacement of the entire patient relative to the beam) were modeled. The cumulative effect of the daily variable and systematic PRE resulted in an absolute mean point dose differences of <0.7% in the three treatment techniques. For daily variable PRE, the interquartile range of point dose differences was small, and within 1% in all techniques. For a systematic PRE, the interquartile range was larger, and within 1.5% for 3DCRT (MC), 1% for 3DCRT (CC), 2% for VMAT (MC), and 2.5% for tomotherapy (CS).

### 3.2 DVH metrics

**FIGURE 5:**
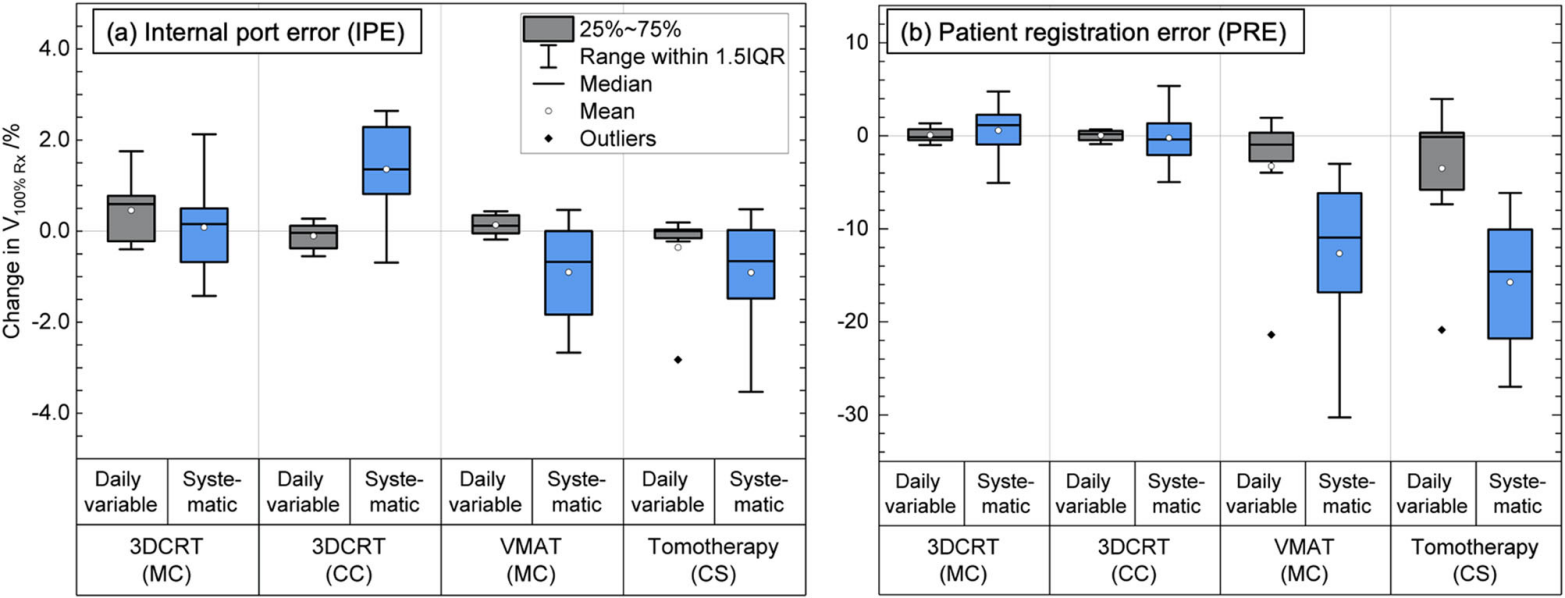
The change in V_100%Rx_ (volume receiving 100% of the prescription dose) of the region of interest (ROI) for all patients when port positional errors were modeled in the three treatment techniques (tangential three‐dimensional conformal radiation therapy [3DCRT], volumetric modulated arc therapy (VMAT), and helical tomotherapy). The modeled daily variable errors are in gray, and systematic errors are in blue. Panel (a) is for internal port error (IPE) and panel (b) is for patient registration error (PRE). The ROI is defined as a 5‐mm expansion around the temporary implant within the planning target volume (PTV). The dose calculation engine used with each treatment technique is shown in brackets

The change in V_100%Rx_ of the ROI when the different scenarios of IPE and PRE were modeled in the three treatment techniques is summarized in the boxplots of Figure 5 for all patients. For the DVH metrics, the overall range of data points is provided when relevant, as opposed to the 1%–99% range given in the previous section.

Figure 5a shows the cumulative effect on V_100%Rx_ of the ROI when port positional errors (IPE) were modeled in each treatment technique for all patients. The cumulative effect of the daily variable IPE resulted in absolute mean changes of <0.5% in the three treatment techniques, with ranges of −0.4%–1.8% for 3DCRT (MC), −0.6%–0.3% for 3DCRT (CC), −0.2%–0.4% for VMAT, and −2.8%–0.2% for tomotherapy. The cumulative effect of a systematic IPE resulted in a mean change of 0.1% (range: −1.4%–2.1%) for 3DCRT (MC), 2% (range: −0.7%–2.6%) for 3DCRT (CC), −0.8% (range: −2.7%–0.5%) for VMAT (MC), and −1% (range: −3.5%–0.5%) for tomotherapy (CS).

The cumulative effect on V_100%Rx_ of the ROI when patient positional errors (PREs) were modeled in each of the three treatment techniques is shown in Figure 5b. The cumulative effect of the daily variable PRE resulted in a mean change of <0.1% (range: −1%–1.3%) for 3DCRT (MC), <0.1% (range: −0.9%–0.7%) for 3DCRT (CC), −3% (range: −21%–1.9%) for VMAT (MC), and −3.5% (range: −21%–4.0%) for tomotherapy (CS). The cumulative effect of a systematic PRE resulted in a mean change of −0.2% (range: −5.1%–4.8%) for 3DCRT (MC), 0.6% (range: −5.0%–5.4%) for 3DCRT (CC), −13% (range: −30% to −3.0%) for VMAT (MC), and −16% (range: −27% to −6.1%) for tomotherapy (CS).

For the slices analyzed in the relevant OARs when daily variable and systematic IPE were modeled, V_20Gy_ of the ipsilateral lung and V_5Gy_ of the heart had mean changes with an absolute mean of less than 0.5%. The cumulative effect of daily variable PRE resulted in changes to V_20Gy_ for the ipsilateral lung of less than 2% in all three techniques. Changes to V_5Gy_ of the heart were less than 0.1% when daily variable PREs were modeled in tangential 3DCRT (MC and CC) treatments, however, for VMAT (MC) and helical tomotherapy (CS), V_5Gy_ had a maximum increase of 6% and 10%, respectively, in a left‐sided breast irradiation.

For the slices analyzed in the relevant OARs, when systematic PREs were modeled, V_20Gy_ of the ipsilateral lung increased by as much as 2.8% for 3DCRT (MC and CC), 2.6% for VMAT (MC), and 2.4% for tomotherapy (CS). For the heart, changes to V_5Gy_ were less than 2% in the tangential 3DCRT treatments (MC and CS); however, for VMAT (MC) and helical tomotherapy (CS), V_5Gy_ increased by as much as 14% and 20%, respectively.

**FIGURE 6:**
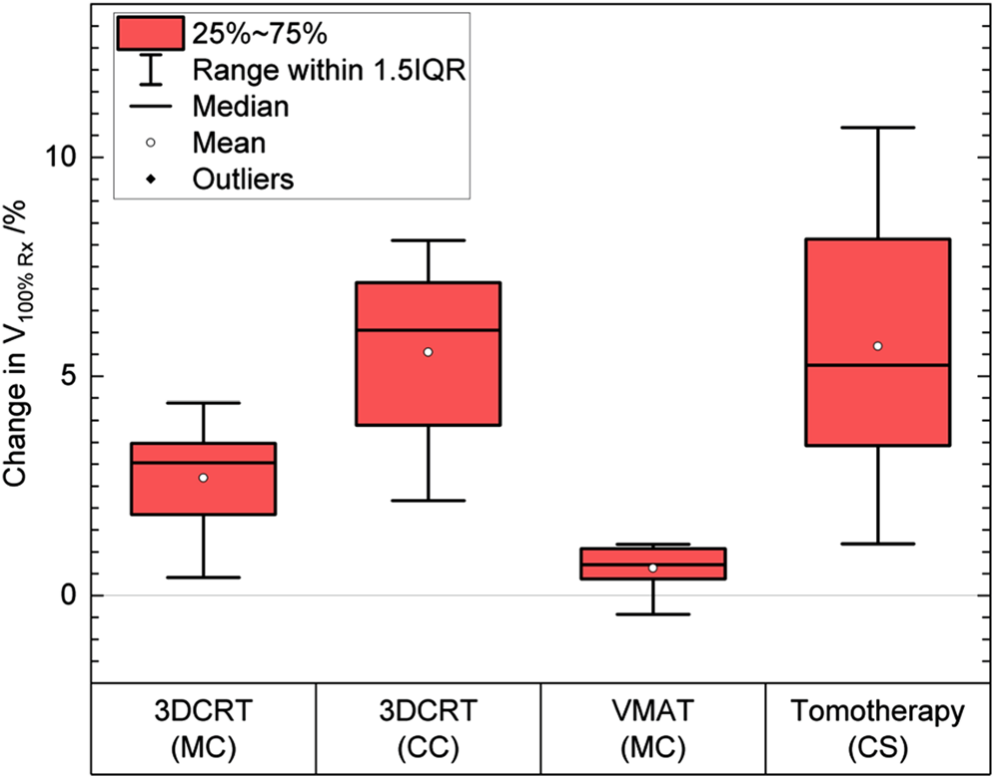
The change in V_100%Rx_ (volume receiving 100% of the prescription dose) of the region of interest (ROI) for all patients when the metal port was overridden with tissue‐equivalent density, relative to the reference dose in the three treatment techniques (tangential three‐dimensional conformal radiation therapy [3DCRT], volumetric modulated arc therapy (VMAT), and helical tomotherapy). The dose calculation engine used with each treatment technique is shown in brackets

For density overrides, the change in V_100%Rx_ of the ROI when the metal port is overridden with tissue‐equivalent density in each of the three treatment techniques is summarized in Figure 6. The mean change in V_100%Rx_ was 2.7% (range: 0.4%–4.4%) for 3DCRT (MC), 6% (range: 2.2%–8.1%) for 3DCRT (CC), <1% (range: −0.4%–1.2%) for VMAT (MC), and 6% (range: 1.2%–11%) for tomotherapy (CS).

## 4 DISCUSSION

The change in V_100%Rx_ of the ROI shows outliers when the daily variable PREs were modeled in the VMAT and helical tomotherapy treatments (Figure 5b). These outliers are not present in the tangential 3DCRT treatment. The origin of these outliers is from two breast cases that had a systematic difference in the position of the metal port throughout the course of the treatment. In tangential 3DCRT treatments, the target coverage is less affected by daily variable and systematic patient positioning errors, as the two main tangential fields are open anteriorly, and the target remains within the radiation field with relatively small patient displacements. In a VMAT or a helical tomotherapy treatment, the delivered dose is more conformal; therefore, the target coverage is compromised to a greater extent when PRE is present. In addition, PRE in VMAT and helical tomotherapy treatments resulted in a greater effect on OARs than a tangential 3DCRT treatment. This can be explained by the differences in the delivery configurations of these three techniques. While the 3DCRT plan contains two half‐blocked parallel‐opposed fields, the VMAT and helical tomotherapy plans consist of many small fields spanning a wider range of angles. Small shifts of the patient relative to these beams can displace a region of the ipsilateral lung and heart in/out the field of radiation.

The cumulative effect of the daily variable and systematic IPE in tangential treatments result in a higher variation of point dose differences in the ROI than in VMAT and helical tomotherapy treatments, as indicated with the 1%–99% range of point dose differences in Figure 5a. From Figure 3a,b (see arrows), it can be seen that there is some dose reduction in the skin in the shadow of the metal port that is outside the non‐biological saline implant, which can be clinically significant because the skin and the chest wall are the possible location for subcutaneous and chest wall recurrences.^16^

In reality, the inter‐fractional positional variations of the metal port arise from a combination of IPE and PRE. The results of this study indicate that VMAT and helical tomotherapy treatments are more robust when patient registration errors are minimized. Therefore, the misalignment of the metal port during patient registration is acceptable when a more optimal anatomy match can be achieved.

Overriding the metal port with tissue density was found to overestimate the dose to the ROI in the three treatment techniques. This is in agreement with previous studies.^9^, ^11^, ^17^ The difference between VMAT and helical tomotherapy treatments in Figure 6 can be attributed to two reasons. First, the beam delivery configuration in the two techniques is different. For all the VMAT plans, the arc was limited to 230°, while helical tomotherapy uses a continuously rotating fan beam, irradiating the target from a wider range of directions. Therefore, ignoring the high‐density metal port in a helical tomotherapy plan may expose a larger volume of the ROI to the beam, thus, overestimating V_100%Rx_ to a greater extent. Another distinction between the two techniques is their dose calculation algorithms. While tomotherapy uses CS, MC was used to calculate the VMAT dose distributions which models the presence of the metal port differently.

The treatment planning kVCT contains heavy metal artefacts which introduce an uncertainty when locating and contouring the metal port during treatment planning. This can result in a systematic error in the true position of the metal port. However, the results of this study indicate that the cumulative dosimetric effect of a systematic IPE on target coverage and OARs is very small in the three treatment techniques. A clinical implication of the above results and from Figure 6 is that contouring the metal port, and assigning it a proper density is an overall better strategy than overriding it with tissue‐equivalent density.

## 5 CONCLUSION

The robustness of tangential 3DCRT, VMAT, and helical tomotherapy treatment techniques against the inter‐fractional positional variations of the metal port of tissue expanders was evaluated. When PREs were modeled, the target coverage and OARs were affected the most in VMAT and helical tomotherapy treatments. The presence of IPE resulted in the highest variation in point dose differences in the target for tangential 3DCRT treatments, suggesting that areas of the skin that fall under the shadow of the metal port can be underdosed. Clinically, the apparent positional deviations of the metal port result from a combination of IPE and PRE, indicating that VMAT and helical tomotherapy are more robust when patient registration errors are minimized. The metal port should be contoured and assigned a proper density during treatment planning, despite the uncertainty involved with accurately identifying the structure of the port.

## CONFLICT OF INTEREST

The authors declare that there is no conflict of interest that could be perceived as prejudicing the impartiality of the research reported.

## AUTHOR CONTRIBUTIONS

Keren Mayorov designed and performed the computational work and data analysis and drafted the manuscript. Patricia Lacasse generated the treatment plans. Elsayed Ali supervised the study, assisted with the study design, interpretation of the results, and drafting the manuscript.
